# Integration of a community-based harm reduction program into a safety net hospital: a qualitative study

**DOI:** 10.1186/s12954-022-00622-8

**Published:** 2022-04-12

**Authors:** Ghulam Karim Khan, Leah Harvey, Samantha Johnson, Paul Long, Simeon Kimmel, Cassandra Pierre, Mari-Lynn Drainoni

**Affiliations:** 1 Section of General Internal Medicine, Department of Medicine, Boston University School of Medicine, 801 Massachusetts Ave, 2nd floor, Boston, MA 02118 USA; 2 Section of Infectious Diseases, Department of Medicine, Boston University School of Medicine, Boston, MA USA; 3 Project TRUST, Boston Medical Center, Boston, MA USA; 4 Department of Infection Control, Boston Medical Center, Boston, MA USA; 5 Evans Center for Implementation and Improvement Sciences, Boston University School of Medicine, Boston, MA USA; 6 Department of Health Law, Policy and Management, Boston University School of Public Health, Boston, MA USA

**Keywords:** Harm reduction, Substance use, Patient-centered care, Addiction consult service

## Abstract

**Background:**

Community-based harm reduction programs reduce morbidity and mortality associated with drug use. While hospital-based inpatient addiction consult services can also improve outcomes for patients using drugs, inpatient clinical care is often focused on acute withdrawal and the medical management of substance use disorders. There has been limited exploration of the integration of community-based harm reduction programs into the hospital setting. We conducted a qualitative study to describe provider perspectives on the implementation of a harm reduction in-reach program.

**Methods:**

We conducted 24 semi-structured interviews with providers from three different primary work sites within a safety net hospital in Boston, MA, in 2021. Interviews explored perceived facilitators and barriers to the implementation of the harm reduction in-reach program in the hospital setting and solicited recommendations for potential improvements to the harm reduction in-reach program. Interviews were analyzed using an inductive approach that incorporated principles of grounded theory methodology to identify prevailing themes.

**Results:**

Twenty-four participants were interviewed from the harm reduction in-reach program, inpatient addiction consult service, and the hospital observation unit. Thematic analysis revealed seven major themes and multiple facilitators and barriers to the implementation of the harm reduction in-reach program. Participants highlighted the impact of power differences within the medical hierarchy on inter-team communication and clinical care, the persistence of addiction-related stigma, the importance of coordination and role delineation between care team members, and the benefits of a streamlined referral process.

**Conclusions:**

Harm reduction programs offer accessible, patient-centered, low-barrier care to patients using drugs. The integration of community-based harm reduction programs into the inpatient setting is a unique opportunity to bridge inpatient and outpatient care and expand the provision of harm reduction services.

*Trial registration*: Not applicable.

## Background

Harm reduction is a collection of practical strategies and ideas designed to decrease the health, social, and/or economic consequences of drug use and serve as an alternative to abstinence-based policies and programming. It acknowledges that people who use drugs have a right to use drugs and to use them in a safe manner. Examples of harm reduction programs include syringe service programs (SSPs), opioid overdose education and naloxone distribution (OEND), and low-barrier supervised consumption sites [1, 2]. Harm reduction is a social movement which seeks to affirm the dignity and rights of people who use drugs. The concept of harm reduction grew from informal, grassroots advocacy efforts led by people who use drugs themselves in response to the widespread moralistic view of drug use during the early HIV/AIDS epidemic [3, 4]. In the USA, the government-sponsored War on Drugs exemplified the antagonistic and punitive response to drug use and addiction. Typically, harm reduction programs function in the community outside of medical centers due to legal and logistical barriers as well as debates about the role of harm reduction in clinical care [5, 6]. Until recently, addiction care and harm reduction specifically had been marginalized or excluded from general medical care, creating further barriers to the integration of harm reduction services and approaches into medical centers [2].

Community harm reduction programs reduce morbidity and mortality associated with drug use and address racial and health-related inequities in a cost-effective manner. SSPs, OEND programs, and supervised consumption, also called overdose prevention sites, have all been shown to lessen the harmful consequences of injection drug use. Harm reduction programs provide education on how to safely inject as well as how to reduce risk of infection [1, 2]. Increased utilization of SSPs and access to naloxone can play a significant role in mitigating the overdose crisis and related outbreaks of infectious diseases [7]. OEND programs have been found to increase readiness for substance use treatment and decrease self-reported opioid use [8]. Likewise, SSPs are effective at reducing HIV transmission when implemented as part of a multifaceted addiction program [9].

Though harm reduction programs have been present in the community for decades, addiction consult services have recently been implemented in inpatient medical settings. Inpatient addiction consult services typically offer clinical care, pharmacologic treatment, behavioral interventions, and patient-centered education for individuals who are hospitalized and use drugs. In contrast to community-based harm reduction programs, inpatient addiction consult services are often focused on the medical management of substance use disorders and acute withdrawal [10]. Limited qualitative data suggest that inpatient addiction consult services can decrease substance use while improving emotional well-being and socioeconomic circumstances [11]. Patients who are seen by multi-disciplinary addiction consult teams while hospitalized are more likely to reduce use of substances in the first month after discharge, as well as a lower 30-day readmission rate [12, 13]. Inpatient addiction consult services are also able to start medications for opioid use disorder as well as link patients to outpatient care [14]. Modeling studies suggest that addiction consult services are cost-effective while also saving lives [15].

Despite the proven benefit of community-based harm reduction programs and high hospitalization rates of people who use drugs (PWUD), there has been limited exploration of the integration of community-based harm reduction programs into the hospital setting. PWUD often experience addiction-related stigma (e.g., poorly controlled withdrawal or pain, loss of autonomy, room searches) during hospitalization and, as a result, delay necessary hospital care and often elect to leave the hospital before they are clinically optimized as a patient-directed discharge [10, 16, 17]. Though inpatient addiction consult services have helped to improve medical management of addiction and withdrawal from substances, integrating harm reduction specialists into the hospital setting could offer a more patient-centered approach to care and could facilitate improved clinical outcomes and safer discharges [10, 18].

In 2019, Project TRUST, a harm reduction program at Boston Medical Center (BMC), initiated an in-reach program in the BMC short-stay observation unit. We conducted a qualitative study with staff from the harm reduction program, observation unit, and addiction consult service (ACS). Our aims were to describe barriers and facilitators to the implementation of a community-based harm reduction in-reach program within the hospital and to solicit suggestions for improvement from the perspectives of both community-based harm reduction specialists and hospital-based healthcare providers.

## Methods

### Study design

We conducted qualitative interviews with 24 individuals working in harm reduction or who have contact with the harm reduction program from the observation unit or ACS. The semi-structured interviews were conducted in order to gain a better understanding of the implementation of a community-based harm reduction in-reach program.

### Study site

The study was conducted at Boston Medical Center (BMC), the largest safety net hospital in New England [19]. BMC is a 514-bed academic medical center with a commitment to providing accessible care to the community. Approximately 72% of BMC patients come from underserved populations, including the low-income and elderly, and the majority are publicly insured. Thirty-two percent of patients do not speak English as their primary language [20]. Thirty-five percent of BMC patients were identified as African-American, 24% as Hispanic or Latino/a, and 25% as white [21]. BMC also serves as a national hub for substance use disorder (SUD) resources. BMC’s comprehensive substance use disorder management includes an office-based addiction treatment program, a low-barrier SUD urgent care clinic, integrated addiction and prenatal care programs, a multifaceted program for adolescents and young adults who use substances, a community-based harm reduction and street outreach program, and an inpatient addiction consult service [22].

BMC’s observation unit is a 28-bed inpatient unit where patients with lower clinical acuity and anticipated length of stay of less than 24 h can be admitted. Patients with skin and soft tissue infections, chest pain and other acute clinical conditions with concomitant substance use disorder are frequently admitted to this unit and may experience acute withdrawal from opioids or other substances. For patients who would not otherwise be admitted to the hospital, the observation unit setting facilitates care coordination beyond what is typically possible in the emergency room and enables access to addiction-related resources that are less readily available in the ambulatory setting. One such resource is ACS, a physician-led inpatient medical consultation team that provides guidance on the intensive medical management of substance use disorders and acute withdrawal, including medication initiation and linkage to outpatient substance use disorder treatment with methadone and buprenorphine, and peer recovery coaching and social work support.

BMC also operates a harm reduction program called Project TRUST (PT). PT is a community-based drop-in site located across the street from BMC with a street outreach team. Through PT, BMC employs harm reduction specialists, a nurse practitioner, and an infectious diseases and addiction medicine physician who provide harm reduction education and safer consumption supplies (e.g., sterile injection and smoking equipment), infectious disease testing and prevention, addiction treatment resources (e.g., detoxification referrals, bridge buprenorphine prescriptions), and offer patient navigation assistance. PT leverages BMC’s expertise in addiction medicine, existing infrastructure, and extensive resources through an integrated prevention and support services navigation model that addresses the barriers to care and services faced by PWUD. Since 2019, PT has also conducted a novel harm reduction hospital in-reach program in the observation unit. PT harm reduction specialists are consulted to come into the inpatient setting to provide harm reduction counseling and facilitate post-discharge linkage to care for PWUD who are admitted to BMC. PT harm reduction specialists advocate to other care hospital-based care teams on behalf of their patients. Both ACS and PT are frequently consulted in the care of PWUD admitted to the BMC observation unit.

### Recruitment

Study participants were individuals who worked in the hospital observation unit, on ACS, or as part of the harm reduction in-reach program. We employed a convenience sampling strategy. An initial recruitment email asking if individuals were willing to participate in a semi-structured, confidential interview was sent to potential participants in each work site via departmental contact listings. Individuals who replied to the recruitment email indicating interest in participating were then contacted by study personnel, and eligibility was assessed according to inclusion criteria: age > 18 years old, job description (nurse, physician, midlevel provider, public health navigator, or social worker), and English speaking.

### Data collection

The semi-structured interview guide (see “Appendix A”) included nine open-ended questions. The initial portion of the guide explored the participants’ understanding of housing insecurity and available support services for patients experiencing housing insecurity admitted to the observation unit. Participants were then asked to describe their perception of the function and value of the harm reduction in-reach program. Subsequent questions explored perceived facilitators and barriers to the use of the harm reduction in-reach program. Participants were specifically asked to describe their department’s support of the harm reduction in-reach program, their impression of the consult referral processes and logistics, educational resources, staff availability, and patient desire for harm reduction services. The interview guide closed with a question regarding the impact of the COVID-19 pandemic on the availability of support services in the hospital and an exploration of any changes to the harm reduction in-reach program that participants would like to see pursued.

Participants completed a written demographic form at the time of data collection. Between January and March 2021, an interviewer (GKK) conducted confidential semi-structured interviews with 24 participants. The institutional review board of Boston Medical Center and the Boston University Medical Campus approved all study protocols and allowed a waiver of documentation of consent by the participants. Interviews were conducted virtually over Zoom Video Communications Inc. (Zoom, Version 5, 2021). Each of the 24 interviews lasted between 20 and 45 min and were audio-recorded with participants’ permission.

### Data analysis

Audio recordings were professionally transcribed verbatim for qualitative analysis. Transcripts were reviewed by the interviewer (GKK) for accuracy. Analysis took an inductive approach that incorporated principles of grounded theory methodology [23]. To create the codebook, two members of the research team (GKK and LH) coded the first transcript independently and met to discuss their findings. Thereafter, the two researchers coded sets of three transcripts independently and met regularly to update the codebook in an iterative fashion. This process continued until all of the interviews had been coded, and consensus had been achieved across all transcripts. All transcripts were coded and analyzed using NVivo 12.0 (QSR International Pty Ltd, version 12, 2018).

Following the completion of the coding process, members of the study team (GKK and LH) engaged in thematic analysis. Emerging themes were identified and discussed by the two coders and then shared with the larger research team for feedback and further discussion using a grounded theory approach [23]. The research team focused on developing themes related to the aims of describing facilitators and barriers to the implementation of a community-based harm reduction in-reach program as well as identifying desired changes to an existing community-based harm reduction in-reach program.

## Results

In total, 24 participants were interviewed. A majority of participants were recruited from ACS [16]. Four participants each were recruited from the community-based harm reduction in-reach program and the observation unit. Most participants self-identified as clinical providers [20], including nurse practitioners, physician assistants, social workers, and physicians. Other participants were identified as harm reduction specialists [4]. Twenty participants were identified as White, two as Black or African-American, and two as Asian. Thirteen participants were identified as female, and 11 participants were identified as male (Table 1).

**Table 1:** Participant characteristics

	N
Gender	
Male	11
Female	13
Race	
White	20
Black or African-American	2
Asian	2
Primary work site	
Observation unit	4
Harm reduction in-reach program	4
Addiction consult service	16
Role	
Clinical provider	20
Harm reduction specialist	4

### Themes

Thematic analysis revealed seven major themes. Participants identified themes ranging from attitudes around the practice of harm reduction in the USA to specific reflections on the community-based harm reduction in-reach program and referral process. An area that was repeatedly discussed was the impact of innate power differences within the medical hierarchy on workplace communication and clinical care. Participants also described the barrier of addiction-related stigma and emphasized the need for the clear delineation of work roles in addiction care. Ultimately, participants expressed the desire to develop accessible, low-barrier harm reduction programs with staffing that reflect the patient populations they serve.

1. The US healthcare system neglects social determinants of health and incentivizes expediency, which often conflicts with harm reduction work.

Interviewees from all three primary work sites identified conflicts between the structure of the US healthcare system and the ethos of harm reduction as barriers to the implementation of a community-based harm reduction in-reach program. A harm reduction worker explained:

I think I have issues with our medical system overall and how – and the for-profit system that we have with insurance and all of that, but you see it really impact those who are the most disadvantaged and need the most resources because there is no flexibility within a lot of these institutions to meet the needs of the patients that have it, have the highest needs. They set systems for those who have the lowest needs and it’s the oftentimes that they’re just making – they’re trying to get these patients that don’t fit the mold to fit the mold instead of changing the mold to fit the patients. – Participant 1

Harm reduction philosophies in healthcare attempt to be patient-centered and focus on the patient’s experience and values. In doing so, the patient’s needs can be prioritized over efficiency and expediency, which could potentially incur a higher financial cost for the health system. For example, a patient might be kept in the hospital beyond when they are “medically” ready for discharge, in order to arrange for an appropriate discharge that addresses housing and safety. The harm reduction specialists, in particular, voiced frustrations and perceived differences in priorities for their patients between the greater health system and their services. One harm reduction worker described:

When I was an outreach worker, and I was doing outreach, I was just pissed all the time because you’re literally just looking at people dying. You’re going why is this just down to money, but a lot of our – the systems in the hospital, a lot of them are established around keeping everything profitable and keeping everything going. That causes a lot of tension. – Participant 1

2. Addiction-related stigma toward patients negatively impacts both patients and harm reduction specialists, contributing to provider burnout and patient mistrust of the medical system.

While the inherent conflict between harm reduction work and system-level financial priorities was frequently implicated as contributing to staff burnout and cited as a key barrier to care, addiction-related stigma that patients face was also described as an important barrier by both ACS providers and harm reduction specialists. This stigma is thought to be perpetuated by a misperception of substance use disorders as a choice or moral failing rather than as a disease process. One addiction consult team member stated:

Yeah, like the way they treat and judge. You know, like the revolving door conversations that they're not going to get no better, you're going to see them again next week. While if you are more educated about the disease of addiction, and what it can do to a person, it's not like that person wanted to grow up to be a person who shoots drugs and overdose. That's not their dreams when they were a little kid, like things happened to them before they even pick up the drug. But they just haven't dealt with and don't know how to deal with and found something to take them outside that pain, and they're running with it. And it's something that became their best friend, their love, their mother, their father, that disease of addiction is there for them to take them out of the pain that they don't want to feel. And the longer they stay out there, the more baggage that comes on top that they have to work to get out of that recover piece. So, if you understand that, okay educated about that, then you will understand that it's not easy. It's not like taking off a Band-Aid and you're cured. – Participant 6

Participants explained that addiction-related stigma is often exemplified by interactions between clinical staff and patients. Negative interactions and micro-aggressions can create an unwelcoming environment for patients, which can disrupt clinical care and potentially prompt patients to leave the hospital. Participants from all three work sites described situations in which stigma has disrupted a therapeutic relationship between patients and providers. An ACS member describes:

People who use drugs are often wary of medical staff and so they often will ask for us, or when they see us, they'll tell us stuff that they wouldn't normally tell, tell the medical staff, whether it be nurses, doctors, social workers, this often may mean, you know, I'm in a lot of pain, but I don't want to say this, because they're gonna think I'm drug seeking, that's something that we get constantly and I hate to say this, but often, I think our clients, and I've done it myself like, they feel overwhelmed or pressured a little bit to say what they think people want to hear. – Participant 15

Addiction-related stigma acts as a barrier to the provision of care to patients by creating a cycle of mistrust that often results in patient-directed discharges. Alternatively, harm reduction programs offer an opportunity to foster trust between patients and the medical system.

3. The inpatient setting and inherent power hierarchies in the clinical environment feel unfamiliar and unwelcoming to community-based harm reduction specialists.

Participants from the harm reduction in-reach program highlighted the difficulties arising from working in a new environment. The harm reduction in-reach program intentionally employs people with lived experience of addiction. One harm reduction worker explained how this intended facilitator of a successful harm reduction program can actually become a barrier:

I think one of the harder things for the outreach workers specifically is they still feel that too, that hierarchy. We really try to hire from the community. Most all of our outreach workers are peers in some sort, so most of them have used substances themselves and had their own struggles with substance use disorder or what have you. What ends up being difficult for the outreach workers is that they don’t get much more respect initially from the providers and nurses on the floor or within the medical – or within medical clinics that we’re going into. The patients can also sense that. – Participant 1

The above participant explains that harm reduction specialists with lived experience are often not treated as professionals with expertise and a valuable skill set. As a result, the harm reduction specialists have found the hierarchical world of inpatient medicine unwelcoming and difficult to navigate.

In addition to the power discrepancies between harm reduction specialists and hospital staff, harm reduction specialists also face an unfamiliar communication style in the inpatient setting.

They understand the hidden curriculum in the OBS unit, which might be for example; if someone is otherwise ready for discharge, an advocate might not be as well received by storming in and saying there's no way you can let this patient out the door right [now]. There's a hidden curriculum that we learn in medical school and residency in terms of how teams interact and make suggestions and disagree with one another. – Participant 4

The hospital environment was viewed as frequently inhospitable to harm reduction specialists who have less experience in the inpatient setting and are less familiar with inpatient workflows and clinical communication.

4. Miscommunication negatively impacts patient care and hinders the provision of harm reduction services.

Participants cited difficulties in communication as a common barrier to the provision of harm reduction services to patients. Harm reduction specialists do not document their conversations and services in the patient chat. One ACS clinician explained that although this issue is not specific to harm reduction, it is an important barrier nonetheless.

So I think that's not a - it's not a problem that's exclusive in any way to these teams. I think it just, whenever you have more - the more people that are involved there's always more risk of kind of poor communication because there are more people who need to communicate, and then there's more risk of harming patient trust or therapeutic alliance by them perceiving kind of lack of team cohesion or lack of communication. – Participant 23

The addition of harm reduction specialists to an already extensive care team necessarily increases and can complicate inter-team communication as well as communication between providers and patients. Any miscommunication can fracture trust and threaten the therapeutic alliance between patients and their care teams.

5. Clearly delineated roles reduce redundancy and streamline harm reduction services.

The potential for miscommunication is compounded when roles are not clearly delineated. The services provided by community-based harm reduction specialists can potentially overlap with hospital-based addiction specialists. One ACS clinician identified the importance of routine and clear task delegation:

I think that it would be helpful to have a daily routine of who to expect at what time, so that those point people will be the point people to talk to if we need to delegate screening for treatment programs or whatever, so that we're not all doing the same thing at the same time. – Participant 11

Clearly defined roles can facilitate the provision of efficient, patient-centered harm reduction services. Many participants also cited a need for a centralized, streamlined process to consult the harm reduction specialists.

6. Harm reduction programs should reflect the patient populations they serve.

In considering the role that a harm reduction team should play in the hospital environment, participants across all three work sites emphasized the specific need for harm reduction specialists to identify with the patient populations they serve. A harm reduction specialist described the in-reach team:

Forming a care team pushes it – pushes them to collaborate and get rid of that hierarchy, so that’s really what we’ve tried to be at Project TRUST is make the outreach workers empowered enough to feel like they can talk up, they can advocate because they are an example for our patients, and if our patients feel like okay, I see somebody looks like me, has the same life experience as me, they are advocating for me. They’re getting what they want. They are able to talk with the doctors like a normal person. It’s not this person that’s just over them that’s smarter than them or doesn’t understand them. – Participant 1

Staffing the harm reduction in-reach program with peers with lived experience facilitates the development of a therapeutic relationship between clinical staff and patient.

While many participants explained that harm reduction specialists with lived experience are best able to provide accessible harm reduction services, several participants further expounded on the ideal for harm reduction specialists to racially and ethnically reflect the populations they serve.

I think having a really diverse group of people in there, I think is helpful, especially patients coming from different backgrounds, people of color, et cetera, I think connecting with people is important in that respect. – Participant 12

7. The referral process should be low barrier and facilitate the identification of patients who would benefit from harm reduction services.

Harm reduction programs work to meet patients where they are, with respect to their substance use. Successful harm reduction programs are flexible in the provision of their services. As one harm reduction specialist explained, each patient has different goals and priorities:

We’ve made our interventions purposely very flexible as far as time periods because we know that, especially with this population that we deal with, they – there is no set formula to getting somebody clean or getting somebody to invest in even caring about themselves enough to use clean needles every day. – Participant 1

Bearing this in mind, one ACS staff member theorized the role that community-based harm reduction specialists could play in the hospital setting:

But I think that integrating community low barrier organizations in our healthcare system could make hospitals much more welcoming places for people experiencing homelessness or with substance use disorders, provide better continuity of care and to places like our bridge clinics that could have ongoing communication with these sort of community-based low-barrier organizations, and help sort of follow people who right now are completely falling through the cracks who get discharged, and we never hear or see from them again, until they're re admitted. – Participant 14

By being more patient-centered than traditional clinical care, community-based harm reduction specialists could help make the hospital a more welcoming environment and interrupt the cycle of stigma and disengagement from care.

When asked about how to design and integrate a harm reduction program into the traditional hospital workflow, participants suggested a variety of models. Suggestions ranged from modeling the service off of a traditional medical consult (e.g., cardiology consult) to the creation of a scheduled “addiction care check-in” for all providers involved in the care of patients with substance use disorders. Participants from all three primary work sites highlighted the need for an automated referral pathway that could identify high-risk patients early in their clinical course in order to involve the harm reduction in-reach program as early as possible and potentially reduce patient-directed discharges.

That could look a whole bunch of different ways. At BMC, we have a lot of the IT infrastructure in terms of the basics in place, but I don't think we've leveraged it as much as we could to do things like have algorithms that might generate an automatic referral. And in a case like this, that might look like someone with a substance use disorder diagnosis on their list who's had two or more OBS presentations or you could hash out what the algorithm would look like, but I think that would be really facilitating so that the Project TRUST team gets pulled in earlier in the admission rather than when if a patient might be feeling like their withdrawal is uncontrolled and not having a good experience and considering leaving in a patient-directed manner. – Participant 4

## Discussion

In this study, a community-based harm reduction in-reach program was identified as a valued program by ACS staff, hospital observation unit staff, and harm reduction specialists. While the need for additional harm reduction services was consistently described, there was a wide variation in perspectives on how to better implement harm reduction services in the hospital. Participants also described multiple barriers and facilitators to the success of a community-based harm reduction in-reach program. These included power hierarchies across hospital systems, addiction-related stigma, and miscommunication within the clinical setting.

Harm reduction specialists specifically described the inherent contradiction between the philosophy and practice of harm reduction and the US healthcare system, which incentivizes patient volume and clinical expediency. They explained that the provision of harm reduction services can include counseling, motivational interviewing, and patient advocacy, which are thoughtful, time-consuming, and often at odds with hospital prioritization of the rapid turnover of beds. However, other addiction clinicians and harm reduction specialists suggested that the overall cost-effectiveness of harm reduction in-reach may outweigh the immediate cost of extending a hospital admission. The existing literature supports this idea and demonstrates that community programs decrease downstream medical complications of substance use [24]. At the same time, costs and overcrowding are often cited as pressures to decrease hospital length of stay and to discharge patients as early as medically feasible [25]. While the financial feasibility of harm reduction in-reach represents an area for additional research, we do know that inpatient addiction medicine consultation is effective at reducing repeat hospitalizations and morbidity associated with substance use disorders and these results suggest that harm reduction in-reach may be a valuable mechanism to support inpatient addiction medicine consultation [12, 26].

Alongside a lack of institutional and systemic support for harm reduction philosophies, participants cited addiction-related stigma as a major barrier to the success of a harm reduction in-reach program. The ACS clinicians and harm reduction specialists interviewed were intimately familiar with the spectrum of addiction care, including harm reduction and non-abstinence-focused strategies. Observation unit staff, however, had less experience with harm reduction. As a result, there were significant discrepancies in participants’ comfort treating patients who use drugs, knowledge of substance use disorder treatment and harm reduction strategies, and consideration of addiction as a disease process. Addiction and harm reduction specialists both emphasized the negative impact of ongoing addiction-related stigma in the hospital setting, which has been demonstrated to be a significant barrier to the implementation of comprehensive harm reduction programs [27]. Multiple participants suggested that negative interactions and perception of stigma could increase patient-directed discharges and disrupt the provision of care, leading to negative health outcomes and hospital re-admissions. Patient-directed discharges represent gaps in our addiction care that could be partially addressed by harm reduction programs and inpatient addiction consult services [17].

The conflict between the ethos of harm reduction and the US healthcare system and ongoing addiction-related stigma contributes to burnout among harm reduction and addiction specialists and negatively impacts PWUD [28]. Harm reduction specialists can potentially bridge these gaps in care and offer an accessible and patient-centered model of care delivery. Harm reduction specialists, who often have lived experience with drug use, continue to refine their expertise by working with clients in the community setting [29]. When these harm reduction specialists enter the hospital setting, they bring those skills and relationships with them. Our results suggest that patients feel much more comfortable when they are able to speak with peers who look like them, come from their communities, and, in some cases, know them from before their hospitalization [30].

Community-based harm reduction specialists typically have limited experience with the structure and workflow of the inpatient setting [31]. Consequently, they struggle to gain acceptance from primarily hospital-based workers and often experience similar stigma as the PWUD whom they are consulted to see [32]. One of the ACS clinicians aptly named the “hidden curriculum” that exists in the hospital. Hospital-based providers undergo years of training that facilitate an effective and efficient form of communication that is medical treatment-focused, rather than harm reduction-focused [33]. Community-based harm reduction specialists are not always privy to this communication style and therefore can be seen as interrupting the efficiency of clinical work flow when they are providing their consultation services to PWUD.

Difficulties in communication highlight the importance of clear role delineation when implementing a harm reduction in-reach program. As many participants described, unclear roles between addiction medicine consultants and harm reduction specialists can be frustrating for both patients and other hospital staff. [34]. However, it is important to note that patients often feel more comfortable communicating their needs and values to the community-based harm reduction specialist, with whom they can more closely identify due to shared experience. [35]. As such, the harm reduction in-reach program may arrive at different clinical conclusions from the addiction consult service. For example, patients may feel pressured to commit to abstinence and pursue pharmacologic treatment for their SUD rather than acknowledging that their current priority is to continue to use. This represents a missed opportunity for harm reduction services, such as safer injection education, and often contributes to a discrepancy between the care plan and patient behaviors. Furthermore, community-based harm reduction specialists who are stigmatized upon entering the hospital environment feel frustrated that they are not being heard or treated as professional colleagues by the hospital-based workers [32].

With respect to the harm reduction in-reach program itself, participants identified the need for the program to reflect the patient population it serves. Our findings are consistent with prior studies proposing that harm reduction in-reach programs should be staffed by peers with lived experience and who are diverse in race, ethnicity, and gender identity [36]. Participants reported that the most positive interactions involving the harm reduction in-reach program occurred when PWUD knew that harm reduction specialists were coming from their own communities.

Participants suggested a wide variety of specific logistical adaptations to better facilitate the integration of community-based harm reduction in-reach programs into the inpatient setting. For example, some participants suggested modeling the harm reduction in-reach referral system after a traditional inpatient specialty service consultation. Many participants cited the benefit of having a separate referral process for the harm reduction in-reach program that is distinct from other addiction services, while maintaining close working relationships between the two teams. While addiction consult services in hospitals often offer counseling and education around safer injection practices, they generally approach patients from a medical perspective rather than with a harm reduction lens [37]. To facilitate the consultation process, participants requested a clear and automated process for involving harm reduction specialists, particularly in the care of high-risk patients who may wish to leave prior to medical optimization. While this need has been described with respect to the identification of patients who would benefit from addiction medicine consults, it was emphasized by participants as a priority for any harm reduction in-reach program [37].

The existing literature focuses on the successes of harm reduction programs in the outpatient setting or on the implementation of inpatient addiction medicine services. This study is unique in that it focuses on the integration of community-based harm reduction programs into the inpatient environment. Because of its qualitative nature, the study is able to explore different perspectives around how to best implement harm reduction in-reach programs, as well as describe some of the potential implications on clinical care. Our results describe important facilitators and barriers to the integration of a community-based harm reduction in-reach program into the inpatient setting. The results augment the existing literature on potential conflicts between harm reduction philosophies and privatized medical systems. Our qualitative analysis reaffirms the continued negative impact of addiction-related stigma, contributing to both provider burnout and patient mistrust of the medical system. This study also highlights the importance of clear communication between harm reduction specialists and inpatient addiction specialists. It adds the voices of both harm reduction and addiction specialists to the literature as they identify ways to improve the provision of harm reduction services to PWUD who are hospitalized.

This study has several limitations. First, our sample size was limited. We recruited from a single site hospital with extensive addiction resources and services that may not be generalizable. Likewise, the majority of participants were clinical providers and the non-clinical perspective is likely underrepresented. However, the heterogeneity of the sample reflects the diverse roles and perspectives of individuals who provide care for PWUD in this inpatient setting. Second, participants were selected using a convenience sample, which may have inadvertently introduced a selection bias of participants who were more supportive of harm reduction hospital in-reach programs.

The morbidity and mortality associated with the ongoing overdose crisis have continued to increase over the past decade. The COVID-19 pandemic further compounded the impact of the overdose crisis by reducing access to SSPs and other harm reduction services and exacerbating underlying mental health issues [38–40]. Harm reduction programs offer a powerful and patient-centered tool to mitigate the negative consequences of substance use [41]. As safe consumption sites and other harm reduction approaches in use in other countries come to the USA, there will be an opportunity and need to integrate these into inpatient settings. The lessons learned from the integration of harm reduction workers into the observation unit may promote effective implementation and mitigate challenges.

## Conclusion

The integration of community-based harm reduction programs into the inpatient setting is a unique opportunity to bridge inpatient and outpatient care and expand the provision of harm reduction services. Future areas for investigation include explorations of patient perspectives on harm reduction in-reach programs as well as the clinical effectiveness and cost-effectiveness of harm reduction in-reach programs.

## Appendix A: Semi-structured interview guide

Interview questions:

1. What clinical problems are unstably housed patients presenting to the BMC Observation Unit with?
2. What is your understanding of support services available for unstably housed patients in the BMC Observation Unit?
3. What is your familiarity with the Project Trust medical outreach team referral program for unstably housed patients in the BMC Observation Unit?
4. What do you think the value of the Project Trust medical outreach team referral program for unstably housed patients in the BMC Observation Unit is, and why do you use this referral program?
5. What is your understanding of how referrals of unstably housed patients in the BMC Observation Unit to the Project Trust medical outreach team program works?
6. What factors do you feel facilitate referrals of unstably housed patients in the BMC Observation Unit to the Project Trust medical outreach team? Facilitating factors may include but are not limited to departmental buy-in, referral processes and logistics, available educational resources, staff availability, staff knowledge of the referral program, Project Trust medical outreach team services, and patient desire for referral.
7. What factors do you feel act as barriers to the referral of unstably housed patients in the BMC Observation Unit to the Project Trust medical outreach team? Barriers may include but are not limited to departmental buy-in, referral processes and logistics, available educational resources, staff availability, staff knowledge of the referral program, Project Trust medical outreach team services, and patient desire for referral.
8. The COVID-19 pandemic has drastically affected our healthcare system. In what ways has it affected the referral of unstably housed patients in the BMC Observation Unit to the Project Trust medical outreach team?
9. How would you like to see the Project Trust medical outreach team evolve and what changes to the referral process for unstably housed patients in the BMC Observation Unit would you like to see?

## Abbreviations

ACS: Addiction consult service
BMC: Boston Medical Center
OEND: Opioid overdose education and naloxone distribution
PWUD: People who use drugs
PT: Project TRUST
SUD: Substance use disorder
SSPs: Syringe service programs
